# The three pillars in treating antibody-mediated encephalitis

**DOI:** 10.1007/s00508-023-02214-3

**Published:** 2023-06-06

**Authors:** S. Macher, G. Bsteh, E. Pataraia, T. Berger, R. Höftberger, P. S. Rommer

**Affiliations:** 1 Department of Neurology, Medical University of Vienna, Vienna, Austria; 2 Division of Neuropathology and Neurochemistry, Department of Neurology, Medical University of Vienna, Vienna, Austria

**Keywords:** Autoimmune encephalitis, Epilepsy, Antineuronal antibodies, Antipsychotics, Immunotherapy

## Abstract

The rapid initiation of immunotherapy has a decisive impact on the course of the disease in patients with antibody-mediated encephalitis (AE). The importance of treating AE with antiseizure medication and antipsychotics is discussed controversially; however, standardized procedures should be ensured, especially for the initiation of treatment in severe disease. Recommendations and guidelines for further interventions in refractory courses are needed. In this review, we contrast the three mainstays of treatment options in patients with AE and attempt to highlight the importance of 1) antiseizure therapy, 2) antipsychotic therapy, and 3) immunotherapy/tumor resection from today’s perspective.

**Supplementary Information:**

The online version of this article (10.1007/s00508-023-02214-3) contains supplementary material, which is available to authorized users.

## Introduction

Autoimmune encephalitides (AE) represent rare neurological conditions. The rising incidence and prevalence indicate an increasing awareness of an underlying organic disorder, especially in presumed primarily psychiatric patients. The last decade has been characterized by an enormous gain of knowledge about symptoms, clinical course, therapeutic options, and prognosis of AE. Nevertheless, all treatment options for AE are off-label and recommendations are based on small studies and expert opinions. Still, it is widely agreed that early initiation of therapy is paramount to improve outcome. Early treatment requires early but also reliable establishment of diagnosis. For this purpose, diagnostic criteria have recently been published [1]. A critical analysis of the possible therapeutic options is essential to assess which measures should be prioritized. In this review, we summarize the existing literature on the therapy of AE and incorporate our personal experience. While there are a variety of reports regarding immunotherapy, those regarding antiseizure and antipsychotic treatment are rare. The following article is divided into three therapeutic mainstays: 1) antiseizure therapy 2) antipsychotic therapy and 3) immunotherapy and tumor treatment.

## Methods

PubMed was searched for the terms “autoimmune encephalitis”: “autoimmune limbic encephalitis”, “autoimmune epilepsy”, Seizures AND “autoimmune encephalitis”, Antipsychotics AND “autoimmune encephalitis”, “NMDAR encephalitis”, “LGI1 encephalitis”, “CASPR2 encephalitis”, “GABA encephalitis”, “DPPX encephalitis”, “GAD encephalitis”, “AMPA encephalitis”, “Glycine encephalitis”. The publication rate on these terms has gradually increased between the years 2000 and 2022 and we focused on clinical cohort studies with a subject number > 20 largely by authors involved in defining the diagnostic criteria for autoimmune encephalitis.

## Results

### Antiseizure treatment

#### Immune-mediated epilepsy

Incidence and prevalence of epilepsy increases in the normal population with age, and patients with increased age are more prone of developing epilepsy [2–4]. In only half of patients aged > 65 years is the etiology of epilepsy identified [5]. Autoimmune etiology was reported in 2.5% of patients (mean age 44 years) treated for status epilepticus in a tertiary center [5]. A small cohort study reported a 4.5% prevalence of antineuronal antibodies (abs) in mainly younger patients with new-onset seizures, whereas a meta-analysis showed a 7.6% pooled prevalence of antineuronal auto-abs in patients older than 16 years with epilepsy of unknown etiology [7, 8].

As a matter of definition, only a fraction of patients develop immune-mediated epilepsy [9]. The International League Against Epilepsy (ILAE) defines immune epilepsy as “an immune disorder in which seizures are a core symptom of the disorder” and “an immune etiology can be conceptualized as where there is evidence of autoimmune-mediated central nervous system inflammation” [10]. The detection of auto-abs and an infectious trigger of epileptic seizures led to the assumption of autoimmune etiology in some epilepsies, resulting in the generation of the term autoimmune epilepsy [11]. Some authors suggested a follow-up interval of 1 year to decide whether to diagnose epilepsy or an autoimmune seizure disorder with seizures in the acute phase that resolves by appropriate treatment [9]. The definition of acute symptomatic seizures comprises the close temporal relationship of the occurring seizure event to any brain injury [12]. The term acute symptomatic seizures secondary to autoimmune encephalitis was suggested when seizures cease after appropriate AE treatment [13]. In this respect, the definition of acute (7 days after onset) seems to be problematic [12]. Therefore, it is important to refine this definition as it affects prescription of antiseizure medication (ASM) and has socioeconomic implications for patients’ lifestyles.

#### Clinical semiology of seizures and electroencephalographic (EEG)-patterns

Seizures are a common clinical feature in patients with AE. The risk of developing seizures was reported at up to 90% in patients with anti-leucine-rich glioma-inactivated 1 (anti-LGI1), anti-gamma-aminobutyric acid A or B (GABA A or B) antibody encephalitis and up to 80% in anti-N-methyl-D-aspartate receptor encephalitis (anti-NMDARE) [14–17]. Seizures may occur at any stage during encephalitis and are frequently observed in limbic encephalitis. In anti-NMDARE seizures as an initial symptom are more frequently observed in males than females [18, 19]. The key point in treating autoimmune seizures is that the use of ASM is usually unsuccessful but seizures mostly resolve by initiation of immunotherapy [15, 20–23]. Especially in patients suffering from anti-LGI1 encephalitis refractory to ASM, striking effects of immunotherapy on seizure freedom were noted mostly within 1 week of initiation [23]. The presence of an underlying neoplasia alters the clinical course in both antibody-mediated and onconeuronal encephalitis, and surgical tumor removal should be sought; however, the question of how long the ASM should be continued is still unresolved.

Regarding seizure freedom a similar timeframe between paraneoplastic and non-paraneoplastic NMDAR, LGI1 or GABA‑B encephalitis was reported after treatment initiation [23]. In anti-NMDARE a relapse rate of 12–30% over up to 2 years was reported [24, 25]. In anti-LGI1 encephalitis relapse rate was reported in up to 35% over 2 years and in 25% of patients suffering from contactin-associated protein-like 2 (CASPR2)-mediated encephalitis [15, 26]. Ongoing disease activity and high relapse risk may be an argument for continuation of ASM. Another argument against termination of ASM may be that patients with mesiotemporal atrophy and/or sclerosis as a sequelae of AE are at higher risk of recurrent seizures. In a population of patients with anti-LGI1 encephalitis it has been shown that nearly all patients develop mesiotemporal atrophy and up to 50% mesiotemporal sclerosis [27]. Given that most patients with anti-LGI1 encephalitis become seizure-free after treatment of encephalitis and discontinuation of ASM, these MRI markers may be of modest predictive value for seizure persistence. In the following, three typical clinical and EEG patterns associated with AE are highlighted; for details on seizure types in AE, please refer to Table 1.

- Pilomotor seizures are observed in limbic encephalitis and temporal lobe epilepsy. They originate in the temporal lobe involving the autonomic structures [28, 29]. Beyond that a frontal origin was reported [30]. In the majority of patients the epileptogenic area was localized ipsilateral to the clinical manifestation site [31]. Piloerection may last only a few seconds and can easily be overlooked by clinicians. As piloerection is often not recognized by patients, clinicians should ask about “goose bumps”. Pilomotor seizures due to AE usually respond well to immunotherapy or in combination with ASM but sometimes also persist [32, 33], which is also our experience. Manifestation of ictal piloerection after diagnosis of new onset focal epilepsy should flag the clinician up to an autoimmune cause [34].
- Faciobrachial dystonic seizures (FBDS) manifest as synchronous puckering of unilateral facial muscles clinically impressing as grimacing together with dystonic posturing of the ipsilateral arm for a few seconds occurring several times per day. FBDS may precede cognitive deficits in patients with anti-LGI1 encephalitis and thus run ahead of the complete manifestation of limbic encephalitis. On the other hand, up to 25% of patients with anti-LGI1 encephalitis predominantly have seizures with only “mild” encephalitis symptoms [23]. The isolated presence of FBDS, unremarkable brain MRI and normal serum sodium levels in early stages of anti-LGI1 encephalitis indicate a circumscribed brain area affected. Hyponatremia is observed in up to 60% of anti-LGI1 patients [15]. The origin of FBDS is presumed to be in the temporal lobe and basal ganglia as illustrated by fluorodeoxyglucose positron emission tomography studies [35]. Also, from the clinician’s view the dystonic postures suggest involvement of the basal ganglia. FBDS are generally considered to occur in non-paraneoplastic encephalitis [36]. These seizure types are poorly manageable by ASM but respond well to immunotherapy, in particular corticosteroids [36–38]. It was observed that the presence of FBDS was associated with progressive cognitive deficits, but patients with anti-LGI1 encephalitis and normal cognition barely harbor anti-LG1 IgG1 antibodies. Early initiation of immunotherapy reduced cognitive deficits and its progression in some patients and led to cessation of FBDS indicating a link and positive effects of early immunotherapy [35, 38].
- Extreme delta brushes (EDB) were defined as rhythmic delta activity with 1–3 Hz with superimposed bursts of rhythmic beta activity with 20–30 Hz. It is considered an ictal-interictal continuum pattern and was primarily observed in severely affected patients with anti-NMDARE [39]. Still, despite severe abnormal EEG findings favorable clinical outcomes were observed in patients [40].

**Table 1:** Autoantibodies – clinical and paraclinical findings

Antibody	Main symptoms	cMRI abnormal*	CSF abnormal**	Seizure semiology [13, 45]	Relapse rate	Overall treatment response
LGI1 [15]	LE, Cognitive/behavioral impairment, peripheral hyperexcitability, seizures, sleep disorder, hyponatremia	++	+	Temporal lobe epilepsy/seizures, FBDS	35%	67% good outcome (mRS 0–2)
CASPR2 [26]	LE, Cognitive/behavioral impairment, cerebellar dysfunction, peripheral hyperexcitability, autonomic dysfunction, sleep disorders	+	+	Temporal lobe epilepsy/seizures	25%	37% full recovery, 52% partial recovery
GABA(A)R [17, 46]	Seizures, SE, Cognitive impairment, movement disorders, psychiatric disorders	++	++	Focal to bilateral tonic clonic seizures (BTCS)	Low	23% full recovery, 64% partial recovery
GABA(B)R [16]	LE, seizures, brainstem dysfunction, movement disorders, CA	++	++	BTCS	21%	37% full recovery, 79% partial recovery
NMDAR [25]	Psychiatric disorders, movement disorders, seizures, cognitive and autonomic dysfunction, speech problems	+	++	Temporal lobe epilepsy/seizures	12%	81% good outcome (mRS 0–2)
DPPX [47]	Hyperekplexia, movement disorders, seizures, cognitive impairment, psychiatric disorders, diarrhea	+	++	Temporal lobe epilepsy/seizures, generalized seizures	23%	60% substantial or moderate improvement
IgLON5 [48, 49]	Sleep disorders, gait abnormalities, movement disorders, bulbar symptoms, cognitive and autonomic dysfunction	+	+	n.a.	n.a.	36–64% mRS 1–3
Gly‑R [50]	SPSD, excessive stimulus-evoked startle, brainstem dysfunction, cognitive impairment	+	+	BTCS	10%	76% good outcome (mRS 0–2)
AMPAR [51]	LE, seizures, mnestic deficits, psychiatric disorders	++	++	Temporal lobe epilepsy/seizures	16%	24% good outcome, 71% at least partial response
GAD65/67 [52, 53]	SPSD, LE, CA, seizures	SPSD + CA, LE ++	++	Temporal lobe epilepsy/seizures	n.a.	70% improvement, no complete recovery

Note: the relapse rate and recovery rate are dependent on and influenced by early initiation and/or escalation of immunotherapy

*CSF* cerebrospinal fluid, *CA* cerebellar ataxia, *LE* limbic encephalitis, *MRI* magnetic resonance imaging, *mRS* modified Rankin Scale, *SPSD* stiff person spectrum disorder, *SE* status epilepticus, *LGI1* anti-leucine-rich glioma-inactivated 1, *GABA (A)R* or *(B)R* anti-gamma-aminobutyric acid A or B, *NMDAR* anti-N-methyl-D-aspartate receptor encephalitis, *CASPR2* contactin-associated protein-like 2, *DPPX* dipeptidyl-peptidase-like protein 6 antibody associated encephalitis, *IgLON5* immunoglobulin-like cell adhesion molecule 5, *GAD65/67* glutamic acid decarboxylase-65 or 67 antibodies, *AMPAR* α-amino-3-hydroxy-5-methyl-4-isoxazolepropionic acid receptor, *Gly-R* anti-glycine receptor antibody

++ > 50%, + < 50%; *findings suggestive of encephalitis

**pleocytosis, intrathecal IgG production, oligoclonal bands

#### Antiseizure medication (ASM) in AE

A cohort study found autoimmune etiology to be responsible for 2.5% of patients hospitalized for status epilepticus (SE) [6]. SE was the most common reason for admission to the intensive care unit (ICU) in patients with AE [41]. In general, there are no evidence-based recommendations for ASM in patients with AE. Small retrospective studies suggested that LEV is among the most commonly used but least effective ASM in AE [23, 37]. For the management of seizures in AE, ASM with sodium blocking abilities (carbamazepine, CBZ, oxcarbazepine, OXC or phenytoin, PHT) are recommended by some authors; however, LEV is a viable medication in terms of dosing and drug interactions and the most frequently used ASM in AE are LEV, CBZ, OXC, valproate (VPA), lacosamide (LCM), LTG and PHT [23].

**Fig. 1:**
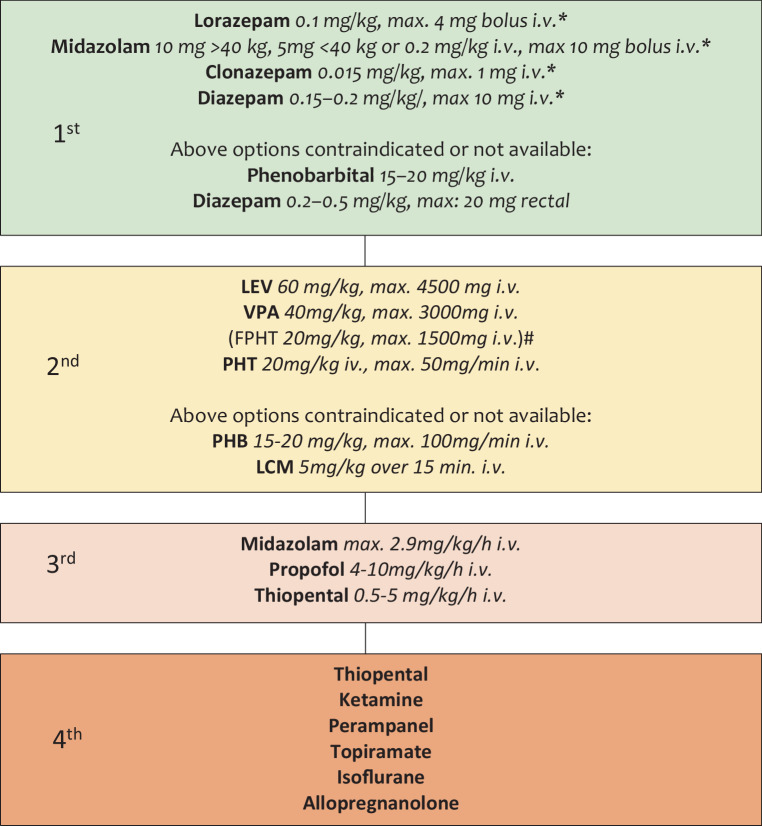
Treatment algorithm for status epilepticus (SE). *Green/1st*: First-line therapy of SE with benzodiazepines, *Yellow/2nd*: First choice treatment of benzodiazepine refractory SE, *Red/3rd*: Treatment of refractory SE, *Brown/4th*: Treatment options of superrefractory SE. *Asterisk* repeat once if necessary, *Rhombus* not available in Austria/Germany

In the following paragraph we refer to the guidelines of the German Society of Neurology and would like to discuss in particular the therapy options in SE (see Fig. 1; [42]). In general, first-line therapy of SE consists of the administration of benzodiazepines, whereby the intravenous (i.v.) administration of lorazepam is most frequently used. Alternatively, diazepam, clonazepam and midazolam can be administered i.v. and in the absence of intravenous access, midazolam can be applied intramuscularly (i.m.) or intranasally. If none of these options are considered or available, rectal diazepam or i.v. phenobarbital can be used. In cases of failure of first line therapy and/or for stabilization after successful first line treatment:

1. LEV can be used as a first choice in benzodiazepine-refractory status epilepticus. A dose of 30–60 mg/kg body weight (max. 500 mg/min., max. 4500 mg cumulative) and an infusion rate of at least 10 min is recommended.
2. VPA can alternatively be used as first choice medication for treatment of benzodiazepine-refractory SE. It is used in a dosage of 40 mg/kg for the therapy of SE (max. 3000 mg infused over at least 10 min.). In further treatment serum concentration of VPA should be monitored and settled in the range of 100–120 ug/l.
3. PHT should be used when VPA or LEV are contraindicated; fosphenytoin is not available in Austria and Germany. PHT should be administered at a dose of 20 mg/kg per min (max. 50 mg/min) through a separate venous line. Plasma PHT levels should settle between 10 and 20 µg/ml and should not exceed 25 µg/ml.
4. PHB and LCM should be considered second-line therapy for SE. PHB is usually administered with 15–20 mg/kg i.v. with a maximum infusion rate of 100 mg/min. LCM is usually administered with 5 mg/kg over 15 min.

For possible medicinal interactions between ASM and relevant drugs for treating autoimmune encephalitis mentioned in this article please refer to supplementary Table 1.

For treatment of refractory SE midazolam (max. 2.9 mg/kg/h i.v.) and propofol (4–10 mg/kg/h i.v.) or thiopental (0.5–5 mg/kg/h i.v.) may be administered. Therapy recommendations in superrefractory SE include topiramate, perampanel, thiopental, ketamine, isoflurane and allopregnanolone. Especially the use of ketamine, which exerts its effects on the NMDA receptor, are discussed controversially in patients with anti-NMDARE but at least some positive treatment effect was reported [43].

In severe courses of AE intercurrent infections are not uncommon. In this context, it should be noted that beta-lactam antibiotics should be used with caution in combination with ASM. This is especially true for patients with renal insufficiency and/or known epileptic seizures. Due to their concentration-increasing or decreasing effect, penicillins, carbapenems and cefepime in particular should be avoided. Fluoroquinolones and macrolides as well as nitrofurantoin and rifampicin should also be used after weighing up possible alternatives and risks [44].

## Antipsychotics

Experience with antipsychotics in patients with AE is limited, and the therapeutic effect is highly controversial. Rapid progression of symptoms usually within few days are so called red flags for AE in patients with first onset psychosis [54]. Especially antidopaminergic agents, often lead to side effects and complications, including the occurrence of malignant neuroleptic syndrome (MNS), in patients with anti-NMDARE [55]. To avoid possible confusion of AE with MNS, some authors advise against the use of highly potent antidopaminergic agents [54, 56]. Instead, benzodiazepines or antipsychotics with a more sedating component, such as olanzapine or VPA should be used [57, 58]. Antipsychotics are usually used early in the course of AE in patients suffering from psychiatric symptoms and when the antibody status is unknown; however, evidence on efficacy of antipsychotics in AE is scarce. Some case reports and small cohort retrospective nonrandomized studies suggested little or no efficacy [55, 59–61]. Nevertheless, one study showed beneficial effects of antipsychotic treatment with amisulpride in patients with first-episode psychosis and serum anti-NMDAR antibodies without immunotherapy. The authors argued that isolated seropositivity does not demand instant immunotherapy and refer to a secondary immune mechanism related to NMDA receptor dysfunction following the glutamate hypothesis of schizophrenia [62]. The question raised is how specific are NMDAR antibodies as they can also be detected in other diseases [63]. In the context of AE there is consensus that only antibodies of the IgG subtype directed against the NR1/2 subunit of the heterotetramer NMDA receptor are pathogenic [64]. Experimentally, using human induced pluripotent stem cells it was shown that receptor internalization occurred independent of Ig subtype [65]. In this context, the presence of antibody subtypes other than IgG in patients with neurological diseases poses both a diagnostic and therapeutic challenge [66]. The prevalence of antineuronal antibodies in schizophrenia has been reported as high as 10% [67]; however, there are some studies with significantly lower prevalence or no antibody detection at all [47–55]. Antibody prevalence especially of the IgG subtype is low in healthy individuals [68, 69]. It was hypothesized that an intact blood-brain barrier (BBB) may prevent circulating serum anti-NMDAR antibodies from migration into the central nervous system. Seroprevalence of antibodies of any isotype (IgA, IgG, IgM) directed against the NR1 subunit of the NMDAR was 8.6% in cohort of schizophrenic patients and 10.5% in the whole cohort of patients including healthy controls and patients with other psychiatric or neurological diseases [70]. In an animal study, these human Igs were injected intravenously into ApoE -/- mice with known BBB leakage and induced behavioral changes independent of Ig subtype. Schizophrenic patients with reported disruption of the BBB (by e.g., brain trauma) and anti NR1 antibodies were shown to have greater symptom severity than those without brain trauma [70]. Also, APoE4 carrier status and presence of serum antibodies against the NR1 subunit were shown to be associated with larger lesion size after ischemic stroke [71]. Still, the fraction of IgG subtype was small in those studies and patients were not classified as having encephalitis. Probably, those antibodies, as part of the natural human reservoir, occur secondarily and are not pathogenic surrogate parameters of disease activity or part of the clearing process after brain damage.

## Immunotherapy and tumor treatment

**Fig. 2:**
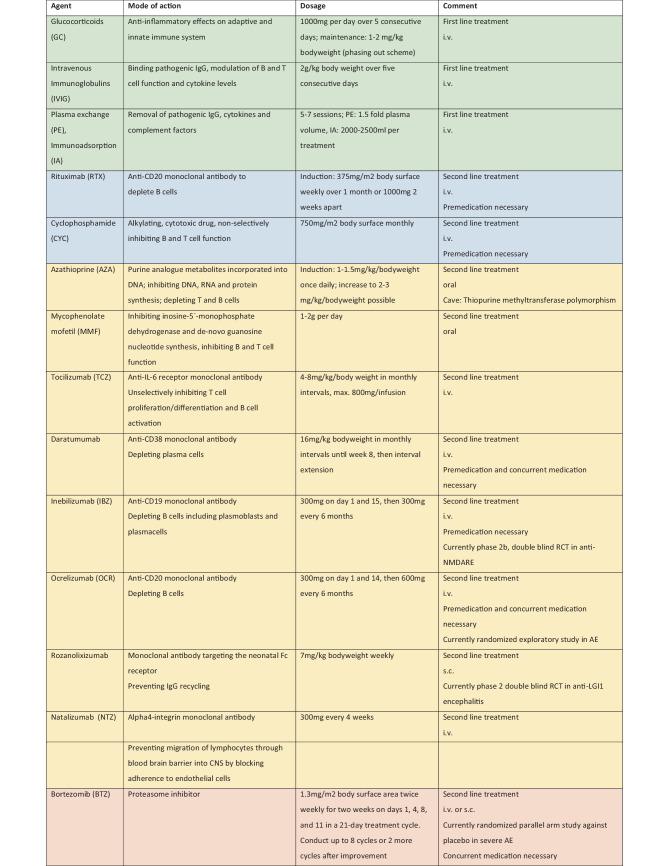
Immunotherapeutic treatment options. *i.v.* intravenous, *s.c.* subcutaneous; *Green*: Standard first line treatment, single use or in combination; *Blue*: Standard second line treatment with the most experience in the treatment of AE in case of failure of first line therapy, single use or in combination; *Yellow*: Other second line therapy options with less experience and future therapy options; *Red*: Therapy escalation to 3rd line therapy with the greatest experience in refractory disease with anti-NMDARE

Targets of treatment in AE are proliferation, differentiation and activation of primarily antibody producing B cells. Different agents are used that either specifically target and selectively (e.g., rituximab, RTX) or nonselectively and indirectly (e.g., tocilizumab) inhibit B cell function, or broadly and nonselectively (e.g., cyclophosphamide, CYC, bortezomib, BTZ) suppress immunological processes. The reduction of B cells and further the circulating antibody load usually lead to an improvement of clinical symptoms. In parallel, a reduction of serum and cerebrospinal fluid (CSF) antibody titers can usually be observed which may serve as biomarkers for disease monitoring. Dormant memory B and plasma cells produce antibodies after they are activated by an antigen. Plasma cells mostly migrate into tissue and bone marrow, persist there and are therefore hard to target by immunotherapeutic agents. The removal of an underlying neoplasm as chronic trigger for the immune system is an early target in treating AE. The mechanistic approach behind refractory antibody-mediated encephalitis and the use of BTZ is to destroy plasma cells that are not or insufficiently attacked by therapies such as RTX and CYC. The following is an overview of immunotherapies for AE (see also Fig. 2). Early initiation of immunotherapy, screening for and resection of an underlying neoplasm have the highest priority in treating antibody-mediated encephalitis [72]. It is associated with a more favorable disease course, faster improvement of symptoms and a lower rate of relapse [25]. The tumor association of antibody-mediated paraneoplastic encephalitides is variable. Based on the antibody present a distinction must be made between low-medium and high-risk types based on the phenotype present and the antibody associated with it. In the presence of high-risk antibodies (e.g., anti-Yo antibodies), tumor screening should be repeated at 6‑month intervals for at least 2 years. In the case of medium-risk antibodies and high-risk phenotype (classical paraneoplastic syndrome, age, smoking history), tumor screening should be repeated at the same interval [73].

When AE is suspected immunotherapy is mandatory and should be applied at low threshold. After exclusion of alternative diagnoses, it is recommended to start with high dose glucocorticoids (GC; 1000 mg methylprednisolone/day i.v. over 5 days) and/or intravenous immunoglobulins (IVIG; 0.4 mg/kg bodyweight/day over 5 days) [19]. After high dose GC, a maintenance dose following a phasing out scheme with oral 1–2 mg/kg bodyweight may be considered. IVIGs were recently studied against placebo in a small randomized trial including patients with epilepsy associated with LGI1/CASPR2 antibodies and showed superiority of IVIG in reduction of seizure frequency [74]. Plasma exchange (PE) and immunoadsorption (IA) represent important and suitable first line treatment options in patients with antibody-mediated AE [75, 76]. In view of their wider availability as well as easier application, IVIG and GC are usually used first, and PE and IA are used subsequently in case of fulminant clinical dynamics or therapeutic failure of the former drugs. In patients with fulminant course of the disease a combination of PE/IA with GC and/or IVIG may be necessary and initiation of second line therapy with RTX or CYC should be prompted. In progressive disease we recommend that therapy escalation to RTX and/or CYC should be done according to clinical judgment but proceeded to at least within 7 days of hospitalization. RTX (1000 mg twice at an interval of 2 weeks or 375 mg/m^2^ body surface weekly over 4 weeks, repeated every 6 months) and/or CYC (750 mg/m^2^ body surface, repeated every 3–4 weeks) should be started in cases of further deterioration despite first line treatment or unresponsiveness after 7 days [77, 78]. It is even rational to escalate to second line treatment if no vital situation prevails but symptom control (e.g., seizures, cognitive deterioration, dysautonomia, movement disorders) is defined as a treatment goal [77, 79].

CYC is an alkylating, cytotoxic agent, which nonselectively depletes T and B cells. RTX is a chimeric monoclonal antibody selectively targeting CD20 + B cells. The treatment effects especially on the levels of antibodies by RTX are to be expected earliest after several weeks [80]. The ability of CYC to cross the BBB is probably lower than 30% [81]. Concentration of RTX in the central nervous system is 0.1–4.4% compared to plasma levels when administered i.v. [82, 83]. Thus, combining RTX and CYC may be beneficial. Other nonselective immunosuppressive treatment options in AE are azathioprine (AZA) or mycophenolate mofetil (MMF) both inhibiting proliferation of B and T cells. AZA depletes B and T cells at higher doses, but preferably B cells at lower dose [84]. AZA is a prodrug, whose metabolites as purine analogues are incorporated into DNA and inhibit DNA, RNA and protein synthesis [78]. AZA is initially administered orally at a dosage of 1–1.5 mg/kg/bodyweight once daily, further dose increase is possible at clinical discretion. Patients with a thiopurine methyltransferase polymorphism are poor metabolizers of AZA and may develop severe myelotoxic adverse effects. MMF inhibits de novo guanosine nucleotide synthesis and is administered orally in a cumulative dose of 1–2g per day. Frequent adverse effects of both MMF and CYC include occurrence of malignancies, hematological disorders and (opportunistic) infections [85]. CYC also has toxic effects on urinary tract, oogenesis, spermatogenesis, and the lungs but is also cardiotoxic and nephrotoxic.

BTZ is a proteasome inhibitor and was FDA approved in 2003 for the treatment of multiple myeloma [86]. To maintain cell homeostasis proteins are tagged by ubiquitin and phosphorylated for degradation in the proteasome. The inhibition of proteasome activity results in inhibition of the transcription factor NF-κB and consequently in apoptosis of the cell, which is also induced by activation of c‑Jun N‑terminal kinase (JNK) and other proteins in tumor cells [87, 88]. BTZ was used as escalation therapy in pretreated anti-NMDARE and is usually administered in cycles on days 1, 4, 8, 11 in a dose of 1.3 mg/m^2^ body surface together with dexamethasone, acyclovir and cotrimoxazole. Each cycle may be repeated up to 6–7 times [89–91]. Adverse effects of BTZ are thrombocytopenia, neutropenia, and fatigue [92]. Besides targeting peripheral blood mononuclear cells, BTZ also accumulates in dorsal root ganglia and peripheral nerve tissue causing BTZ-induced neuropathy (BiPN) [93]. BiPN manifests predominantly as sensory axonal neuropathy, whereas discrepancy between nerve conduction velocity studies and clinical manifestation may occur due to small fibre damage [94]. BiPN is usually reversible and improvements are observed 3 months after discontinuation of BTZ. Whether the combination of BTZ with other agents enhances neurotoxic effects is discussed controversially but may be a limiting factor [95–97].

Tocilizumab (TCZ) is a humanized monoclonal antibody against interleukin (IL) 6 receptor, thereby nonselectively inhibiting proliferation and differentiation of T cells and activation of B cells. TCZ was used as escalation therapy in RTX-refractory AE as reported by retrospective studies and case studies [98–100]. Before using TCZ a latent tuberculosis or hepatitis virus infection needs to be excluded. Monitoring of liver and blood parameters, and cardiovascular function is necessary. Risk of developing malignancies is increased by TCZ and has to be monitored [101].

Daratumumab is a human monoclonal antibody targeting the receptor and adhesion molecule CD38. Plasma cells do not express CD20 and are therefore not depleted by anti-CD20 agents. CD38, on the other hand, is abundantly expressed by plasma cells. There are only case reports on beneficial therapeutic effects of daratumumab in patients with AE [102, 103]. The therapeutic extension to daratumumab is essentially based on the fact that plasma cells keep the inflammation ongoing after B cells have already been depleted by previous therapies. Infusion-related reactions including bronchospasm and laryngeal edema were observed in association with daratumumab. Infections, hematological alterations, sensory neuropathies, cough, diarrhea, and fatigue are other frequently observed adverse effects.

Natalizumab (NTZ) is a humanized monoclonal antibody directed against alpha4-integrin on lymphocytes, thus preventing adhesion to BBB endothelial cells and further trepassing into the CNS [104]. It is approved for relapsing-remitting multiple sclerosis. NTZ reduced seizure frequency but not cognitive impairment in a patient with anti-GAD AE [52].

## Future perspectives

Based on currently ongoing studies registered at clinicaltrials.org, the following section provides an overview of possible future treatment options. These studies primarily seek to compare efficacy of first line treatments and provide a basis for future treatment decisions.

IVIG and GC are considered equally effective as first-line therapy in patients with AE, but efficacy has not been studied head-to-head. There are currently ongoing prospective randomized controlled studies to investigate the effect of early PE versus IVIG combined with GC [105]. The effect of IVIG is further examined in a prospective single arm study in patients with AE [106]. In patients with acute psychosis and evidence of serum or CSF autoantibodies a randomized placebo-controlled trial explores the treatment effect of IVIG (2 g over 2–5 days) combined with RTX (1 g twice at an interval of 14 days) compared to placebo [107]. Another prospective trial focuses on the combination of 10 IA sessions followed by RTX weekly over 4 weeks in pediatric patients with anti-NMDARE [108].

Inebilizumab (IBZ) is a humanized monoclonal antibody directed against the B‑cell surface antigen CD19 and was recently EMA-approved in neuromyelitis optica spectrum disorders [109]. By targeting CD19 instead of CD20 IBZ additionally depletes CD20 negative plasmablasts and plasma cells. IBZ is currently under investigation in patients with anti-NMDARE, who will receive first line immunotherapy and IBZ vs. first line immunotherapy and placebo [109, 110]. A randomized, double-blind, placebo-controlled study examined the efficacy of ocrelizumab (OCR) in patients with AE and antibodies against NMDAR, LGI1, CASPR 2 or dipeptidyl-peptidase-like protein 6 antibody-associated encephalitis (DPPX). OCR is a humanized monoclonal anti-CD20 antibody approved for treatment of relapsing and primary progressive multiple sclerosis [111]. Rozanolixizumab is a monoclonal antibody targeting the neonatal Fc receptor preventing IgG from recycling thereby reducing pathogenic IgG antibody load. The agent is compared to placebo in a parallel arm design with seizure freedom as the primary endpoint in patients with anti-LGI1 encephalitis [112, 113].

Subcutaneous interleukin 2 (IL-2), whose hypothesized mechanism of action is ameliorating inflammation by the upregulation of regulatory T cells, is currently studied in patients with treatment refractory AE administered over 9 weeks [114]. A prospective parallel arm assignment investigates the efficacy of BTZ vs. placebo in patients with severe AE [115]. The effects of transcranial direct current stimulation on synaptic plasticity in patients with anti-NMDARE, aiming to improve NMDAR function and neuronal signalling, is currently investigated by using transcranial magnetic stimulation and motor tasks by a German study group [116].

## Conclusion

Seizures and psychiatric symptoms are among the most common symptoms of patients with AE. Seizures may be typical for limbic encephalitis (FBDS, pilomotor seizures), but there is no pathognomonic clinical or EEG pattern. The same is true for psychiatric symptoms, which may be predominant or isolated during encephalitis. For identification of patients suffering from AE or paraneoplastic encephalitis clinical criteria should be applied but aggravatingly, unremarkable brain MRI, CSF or EEG do not exclude AE [1, 73, 117]. Usually, an epileptic seizure leads to further diagnostics in primarily psychiatric patients and to initiation of ASM in patients with suspected encephalitis. As epileptic seizures often occur before the antibody status has been determined or diagnostic criteria can be applied, the question of whether ASM therapy should be initiated is usually unnecessary. Consistent with the current literature, we see immunotherapy as the determining factor in the therapy of patients with AE. We recommend that immunotherapy is considered mandatory in treating AE and that, if clinically indicated, concomitant antiseizure medication is applied and escalated if clinically necessary. Antipsychotics are more likely to be used at the onset of an illness when the cause is unclear and should be considered optional due to the lack of evidence of efficacy in AE and possible side effects worsening the clinical picture. In practice, all three mainstays will probably be applied, but the special importance of immunotherapy should be emphasized.

### Supplementary Information

Supplement Table 1: Possible medication interaction between ASM and relevant drugs mentioned in this article to treat autoimmune encephalitis [118]
