# Supplementary material for: The three pillars in treating antibody-mediated encephalitis
Source: Wien Klin Wochenschr. 2023 Jun 6;136(1-2):13–24. doi: 10.1007/s00508-023-02214-3 (PMC10776469; doi:10.1007/s00508-023-02214-3)
Supplement: Supplementary file 1 — Supplement Table 1: Possible medication interaction between ASM and relevant drugs mentioned in this article to treat autoimmune encephalitis [118] [file 508_2023_2214_MOESM1_ESM.docx]

| Drug | Possible Interactions |
| --- | --- |
| LEV | PL↑: PHB, CBZ, PHT, MTX  LTG may increase clearance of LEV |
| VPA | VPA PL↑: Clobazam, Fluoxetine, Sertralin  VPA PL↓: LCM, LTG, PER, PHB, PHT, CBZ, Meropenem, Imipenem, MTX, Aciclovir  VPA may influence plasma levels of:  PL↑: Amitryptiline, Quetiapine, PHB, CBZ, non-protein bound PHT, Lorazepam, Propofol (reduced dose necessary for sedation)  PL↓: Olanzapine |
| PHT | PHT PL↑: Diazepam, Clarithromycin, Trimethoprim, Fluoxetine, Trazodone, Risperidone  PHT PL↓: Ciprofloxacin, Aciclovir  PHT PL ↑↓: CBZ, PHB, VPA, Clonazepam, Dexamethasone  PHT may influence plasma levels of:  PL↑: Clobazame  PL↓: Quetiapin, Risperidone, Clonazepam, Diazepame, Midazolam, Dexamethasone, Methylprednisolone, Prednisolone, CBZ, CYC, MTX |
| CBZ | CBZ PL↑: Quetiapine, Risperidone, Clobazam, VPA  CBZ PL↓: PER, PHT  CBZ may influence plasma levels of:  PL↑: Clobazam  PL↓: CYC, MTX, Amytryptiline, Citalopram, Apriprazol, Olanzapine, Quetiapine, Diazepam, Clonazepam, Midazolam, Dexamethasone, Methylprednisolone, Prednisolone |
| LTG | LTG PL↑: Sertraline, VPA  LTG PL↓: Olanzapine, Apriprazole, CBZ, PHB PER |
| PHB | PHB PL ↑: PHT, VPA  PHB may influene plasma levels of:  PL ↑: clobazam  PL ↓: MTX, clonazepam, dexamethasone, methylprednisolone, prednisolone, MTX, CBZ |
| PER | PER PL↑: VPA  PER PL ↓: CZB, PHT, PHB  PER may influence plasma levels of:  PL↓: Midazolam CBZ clobazam |
| LCM | PHB, PHT, CBZ may decrease LCM plasma levels |

Supplement Table 1: Possible medication interaction between ASM and relevant drugs mentioned in this article to treat autoimmune encephalitis. PL ↑: may increase plasma levels; PL↓: may decrease plasma levels; PL↑↓: may in- or decrease plasma levels. PHB phenobarbital, CBZ carbamazepin, PHT phenytoin, LEV levetiracetam, VPA valproate, LTG lamotrigin, PER perampanel, LCM lacosamid, MTX methotrexate, CYC cyclophosphamide (119)
